# Parents’ Preferred Age (9–12) for HPV Vaccination: Decision-Making and Rationale

**DOI:** 10.3390/vaccines14050422

**Published:** 2026-05-07

**Authors:** Holly B. Fontenot, Siobhan Coad, Erica J. Liebermann, Erika L. Thompson, Emma Collo, Tiffannie Chang, Melanie Kornides, Gregory Zimet

**Affiliations:** 1 School of Nursing and Dental Hygiene, University of Hawaii at Manoa, Honolulu, HI 96822, USA; 2 Thompson School of Social Work & Public Health, University of Hawaii at Manoa, Honolulu, HI 96822, USA; sgcoad@hawaii.edu; 3 College of Nursing, University of Rhode Island, Providence, RI 02903, USA; eliebermann@uri.edu; 4 Kate Marmion School of Public Health, University of Texas at San Antonio, San Antonio, TX 78229, USA; thompsone1@uthscsa.edu; 5 College of Nursing, Creighton University, Omaha, NE 68178, USA; 6 School of Nursing, University of Pennsylvania, Philadelphia, PA 19104, USA; kornides@nursing.upenn.edu; 7 School of Medicine, Indiana University, Indianapolis, IN 46202, USA; gzimet@iu.edu

**Keywords:** human papillomavirus, HPV, parent decision-making, age of vaccination

## Abstract

**Background/Objective**: The objective of this study was to explore parental preferences for the age of HPV vaccination (9–12) and the rationales for these preferences. **Methods**: Four online text-based focus groups were conducted with a national sample of 43 parents who have at least one child aged 9–10 years. Participants discussed preferred age for HPV vaccination and how it relates to the routine adolescent vaccine schedule in the United States (US). Content analysis was utilized to identify emergent themes. **Results**: Three themes surrounding parents’ motivating factors related to HPV-vaccination schedule preferences emerged from the analysis of the focus group discussions: (1) a belief that age 9 is too young versus a belief in early protection, (2) the number of shots administered per visit (a desire to spread shots out or group together), and (3) the parent follows their health care provider’s recommendations. **Conclusions**: This qualitative study of parental preferences regarding HPV vaccination age and how it relates to the routine adolescent vaccine schedule reveals mixed parental decision-making and rationales for vaccine acceptance and at which age. Given the dynamic vaccine policy landscape in the US, it is essential for providers to understand parental perspectives and motivating factors related to vaccine decision-making and integrate these drivers into clinical practice to best support families and public health at large.

## 1. Introduction

In an effort to improve human papillomavirus (HPV) vaccination rates in the United States (US), given the suboptimal uptake as compared to other routinely recommended adolescent vaccines [1], national organizations (specifically the American Cancer Society [ACS] and the American Academy of Pediatrics [AAP]) have promoted starting the routine recommendation for HPV vaccination at age 9 years [2,3]. The current routine recommendation for the HPV vaccine set by the Centers for Disease Control and Prevention (CDC)/Advisory Committee on Immunization Practices (ACIP) is age 11–12 years [4]. Part of the rationale for beginning recommendation at age 9 includes the potential for: (1) more time for completion of the series by age 13, (2) a strong immune response, (3) more opportunity to vaccinate before first exposure to HPV, (4) a decrease in the number of shots administered per clinic visit, which may be preferred by some parents and children, and (5) an increase in HPV vaccination rates [3].

Several papers have been published about the potential advantages of starting HPV vaccination at age 9 or 10 [5,6,7,8], however the empirical evidence is mixed [9]. Several recent reports have shown that the age 9 initiation for HPV vaccination has resulted in improved on-time HPV vaccine completion [10,11,12]. Furthermore, some health care providers find this transition to recommending the vaccination at age 9 acceptable [13,14,15]. In addition, some clinics and health plans are willing and able to implement and/or support HPV vaccination at age 9 or 10 [16,17]. At the same time, some providers have reported that age 9–10 for administering HPV vaccination did not alter parental questions or concerns about vaccination and did not impact the amount of time required for discussion about the vaccine [15].

Of note, no research published to date has identified whether an age 9–10 recommendation would translate to an actual increase in HPV vaccine coverage. The research on parental intentions and attitudes is mixed, as emergent research on parents’ perspectives demonstrates that a transition to age 9 for HPV vaccination is not necessarily straightforward. Coad et al. (2026) reported that lowering the recommended age of HPV vaccination from ages 11–12 to 9–10 largely did not change parents’ thoughts about obtaining the HPV vaccine for an unvaccinated child [18]. In contrast, Saxena et al. (2025) reported that 70% of their sample of parents with an HPV un-vaccinated child (aged 11–12) (N = 200) most likely would have vaccinated their child at age 9 or 10, if recommended by their child’s health care provider [19]. Similarly, in another study published by Saxena et al. (2025), 90% of parents with children vaccinated at ages 11–12 indicated that they would have accepted HPV vaccination at an earlier age [20]. In contrast, Zimet et al. (2025) found that parents had mixed viewpoints, and approximately half preferred HPV vaccination at age 11 as compared to vaccination at age 9 or 10 [21].

To expand on the current literature related to parental preferences for the age of HPV vaccination and rationales for these preferences, we conducted focus groups with parents of children ages 9–10 to elicit their perspectives. Qualitative data allows for the discovery of factors influencing parental decision-making and may inform health care provider communication about evolving vaccine recommendations.

## 2. Methods

### 2.1. Procedures and Study Population

This national qualitative focus group study, conducted in June of 2025, used an online synchronous and interactive text-based focus group method to explore parental perspectives about receiving an HPV vaccine recommendation from their health care provider at varied ages (9–12). This research methodology has been used extensively in vaccination and sexual health research as it reduces barriers to participation, promotes safety in expressing divergent perspectives, and facilitates geographic and sociodemographic diversity of the sample [22,23,24]. Eligibility criteria included: (1) parent of a child aged 9–10; (2) resident of the US; (3) able to read and write in English; and (4) access to the internet and an electronic device necessary for participation (computer, tablet, etc.).

Potential participants were recruited from an existing US national research panel operated by InsideHeads, LLC (St. John, VI, USA), an experienced marketing research company. The target sample size was between 40 and 45 to ensure data saturation. InsideHeads emailed potential participants from their national panel a link to the secure research website that included the study information and determined the potential participant’s eligibility. InsideHeads then emailed eligible participants a copy of the study-specific informed consent and provided instructions on how to attend one of the four focus groups (including date/time and login info). All participants were assigned a random pseudonym as they logged into the study-specific secure web-based qualitative engagement platform from their personal computing devices. Participants were able to participate in a location of their own choosing. As participants joined the synchronous online text-based discussion room (similar to an online chatroom), they were first welcomed by the principal investigator and then provided with a link to a brief demographic questionnaire (Qualtrics^®^ XM Platform survey software, June 2026) that also re-affirmed study consent. Once that step was completed, participants were re-welcomed to the discussion room by the principal investigator who moderated all focus groups. All discussions were also observed by three additional members of the research team who recorded field notes. Participants actively engaged in real-time dialog by typing their comments, beliefs, and responses (to both the moderator and each other) into the platform. Participants had the option to use emojis (for example, ☹) to communicate emotions non-verbally. All data collected remained anonymous and de-identified and, InsideHeads, per their protocol/standard renumeration practices, sent participants a $100 electronic gift card as compensation for their participation in the 1 h discussions. The study protocol and procedures were approved by the University of Hawaii at the Manoa Institutional Review Board.

### 2.2. Measures

Quantitative data (demographic data) were collected via Qualtrics and included: participants’ age, identified sex, race/ethnicity, education level, geographic location (urban, suburban, or rural), and US region (West, Midwest, Southwest, Northeast, and Southeast). Additionally, data were gathered on family constitution (number and age of children in household) and the HPV vaccine status of both the participant and their child/ren. Parents’ perceived importance of childhood vaccination was measured using a single item: “How important is it that your child(ren) receive all of their recommended vaccines?” Responses were collected using a 3-point Likert Item including “very important,” “important,” and “not important.”

Qualitative data (focus group data) were elicited using a semi-structured interview guide developed by the study team. Parents were first asked to share their perspectives on the HPV vaccine (generally and specifically related to age of initiation). Next, and to establish a shared baseline understanding among participants, they were informed of the June 2025 routine adolescent vaccine schedule (vaccines routinely recommended at ages 11–12) set by CDC/ACIP (i.e., HPV, tetanus, diphtheria, and acellular pertussis [Tdap] and meningococcal ACWY [MenACWY]) [4] as well as expanded HPV vaccine age recommendations (beginning at age 9) by other professional organizations (ACS and AAP) [2,25]. After gathering further impressions and perspectives about age for HPV vaccination recommendation in particular, participants were shown three potential adolescent vaccine schedule scenarios, and each was color coded (see Table 1). Participants were asked to provide thoughts on each of the following potential options for their child: (1) first dose of HPV at age 11, administered with Tdap and MenACWY, with the second dose at age 12 (color coded blue); (2) first dose of HPV administered at age 9 with the second dose at age 10, followed by Tdap and MenACWY at age 11 (color coded orange); and (3) first dose of HPV administered at age 10, followed by second dose at age 11 with Tdap and MenACWY (color coded green). First, participants discussed the pros and cons of each potential option one at a time, then participants engaged in a reflective and summative discussion, and finally each individual identified their most preferred vaccine schedule and provided their reasoning for their choice.

### 2.3. Analysis

Quantitative data management and analyses were conducted in Microsoft Excel. Descriptive analyses included the calculation of percentages for all categorical data. The quantitative survey data was used only to provide context for the qualitative findings. Qualitative text data were downloaded from the secure study-specific online discussion portal directly into Microsoft Word. The qualitative data were also uploaded into a separate Microsoft Excel database for qualitative data management and qualitative analysis (the investigators use Excel to organize, visualize, and code data by hand). Qualitative inductive content analysis was conducted by four members of the research team, including the principal investigator. The team first immersed themselves in the data (reading and re-reading the text) and conducted initial coding independently, then met iteratively to discuss emergent first-level codes, concepts, and themes. Following consensus on codes and themes, data were independently re-coded. Research team members continued to meet weekly to discuss the analysis, ensure consistency across coders, resolve discrepancies, and reach consensus on any conflicting interpretations. As the research team values reflexivity, a negotiated agreement process leading to consensus was employed consistent with constructivist approaches to qualitative research [26]. Initial codes were used to develop broad categories, from which the final themes and sub-themes emerged. In addition to the thematic analysis, some count data were abstracted from the qualitative data set to further elucidate and add clarity to the qualitative findings.

## 3. Results

### 3.1. Quantitative Results

Four online focus groups were conducted (each 60 min) with a total sample size of 43 participants (10–12 per group). Table 2 highlights participant characteristics. Of these participants with a 9–10-year-old child, nearly half (n = 20, 46.5%) also had a child/children aged 11 years or older. Participants included both women (n = 27, 62.8%) and men (n = 16, 37.2%) ranging in age from 29 to 53 years, and were dispersed across the following five US Regions: West (n = 9, 20.9%), Midwest (n = 11, 25.6%), Northeast (n = 11, 25.6%), and Southeast (n = 12, 27.9%); however, no participants lived in the Southwest. Due to late arrival to the online discussion, some demographic information could not be collected for one participant. As such, the following reflects the 42 participants for whom demographic information was obtained. The majority were non-Hispanic white (n = 27, 64.3%), lived in suburban areas (n = 22, 52.4%) and had a college degree or higher (n = 30, 71.4%). Most (n = 28, 66.7%) had not been personally vaccinated for HPV as an adolescent or young adult. Few participants (n = 6, 14.3%) had already vaccinated their 9–10-year-old child for HPV, and most participants with older children had previously vaccinated their older child/ren for HPV (n = 17, 85%). When asked how important it was that their children receive all recommended vaccines, most responded, “very important,” (n = 26, 61.9%) or “important,” (n = 13, 31%).

In addition to the demographic survey data, some count data were abstracted from the qualitative dataset. The majority of participants reported a positive attitude towards obtaining the HPV vaccination for their child at some point (n = 32, 74.5%). The remaining parents were categorized as hesitant (n = 7, 16.2%) or against the HPV vaccine (n = 4, 9.3%). Participants had mixed preferences towards timing for HPV vaccine initiation for their child(ren). When directly asked, nearly half (n = 20; 46.5%) stated that they preferred the idea of vaccine initiation early, at age 9. The other half (53.5%) preferred initiating at age 11 (n = 12; 27.9%), age 10 years (n = 9; 20.9%), or did not indicate a preference (n = 2; 4.7%). See Supplement Table S1 for parental characteristics by preferred age to initiate HPV vaccination.

### 3.2. Qualitative Results

The qualitative results elucidate the parental decision-making and rationale behind their preference for age to initiate the HPV vaccine. Participants’ discussion and candid reactions to each of the three potential vaccine schedules revealed important insights into the factors influencing their decision-making about the HPV vaccination. As the three schedules were presented in succession, parents’ preferences commonly shifted upon seeing subsequent options. At the end and with their reflection, parents selected their most preferred schedule/age for HPV vaccination initiation and voiced their underlying priorities/motivating factors/rationale that anchored their decisions. Two parents who did not select a favorite option were excluded from the thematic generation. The major themes guiding schedule preference/age for HPV vaccination were: (1) a belief that age 9 is too young versus a belief in early protection, (2) the number of shots administered per visit (a desire to spread shots out or group together), and (3) the parent follows their health care provider’s recommendations, whatever that may be (see Table 3 for qualitative illustrative quotes highlighting themes; plus Supplement Table S2 which depicts primary rationale for preference regarding age for HPV vaccine initiation using count data from the qualitative data set).

#### 3.2.1. A Belief That Age 9 Is Too Young Versus a Belief in Early Protection

More than any other factor, the specific age of vaccine initiation determined participants’ schedule preferences. The belief that age 9 is too young for HPV vaccine initiation was the most frequently cited driver for schedule preference among parents (n = 15), and most parents with this belief (n = 9) favored vaccination at ages 11–12. Participants stated, “I would choose blue [initiating at age 11]. I’m just not sure I think that my 9-year-old would need this shot at such an early age” (Female, age 40), “I prefer the blue one [initiating at age 11]. Too young at 9 and at least you get to space it out [first to second dose] a little between 11 and 12 years old,” (Female, age 46) and “I like the blue [initiating at age 11] it’s easier to get an older child shots” (Female, age 34). 

Comments around sexual activity and development often accompanied the above noted preferences. Two participants shared, “This is all about sexually active children and the thought that 9 is beginning of this first potentially coming into contact with my kid is unthinkable for me,” (Female, age 46) and “I think it’s too soon [for the HPV vaccine at age 9]. Especially for a girl… their body is still developing” (Male, age 43).

Participants (n = 6) who preferred younger initiation due to beliefs in the value of early protection favored both the initiation at age 9 (n = 3) and age 10 (n = 3) options in equal numbers. Participants who favored initiation at age 10 noted, “I’m for [age 9 or 10] if we can protect the kiddos at a younger age” (Male, age 45) and, “I like the idea of potentially getting added benefits of vaccinating earlier,” (Female, age 37). Another stated, “I prefer getting them out of the way earlier to avoid exposure” (Female, age 45). The discussion of sexual activity also arose in relation to early protection, “I would pick orange [initiation at age 9] because the HPV schedule is complete at age 10… BEFORE they tend to think of/or engage in sexual activity” (Male, age 39).

#### 3.2.2. The Number of Shots Administered per Visit (A Desire to Spread Shots out or Group Together)

Participants’ schedule preferences were informed by their opinions toward the number of shots their child would receive at each visit (n = 10). A desire to spread shots over multiple visits motivated many parents to favor the age 9 scenario (n = 8). Participants noted, “I think it would be good to break them up [shots] a bit, so they [don’t] have to get the 3 [shots] at age 11,” (Female, age 42), and “I like breaking them up more as long as they are still effective… I know it is 3 visits but we are going anyway. I like the idea of breaking up the shots a little bit” (Female, age 43). Lastly, one said, “I would want the three vaccines to be spread out a bit to allow my child recover from them” (Female, age 37). 

Some who were motivated by spacing out vaccines across multiple visits considered choosing age 9 to start even though they viewed it as too young. They wondered instead if they could pick a different schedule where they delayed vaccination to after age 11. For instance, “I like this schedule [initiation at age 9], but it can start at maybe age 13, finish by 16?” and “Break up [the vaccines administered] at age 11… Give [shots at] 11, 12, 13, and 14 [years of age] … Push the vaccines later and spread them out” (Male, age 36). One noted a concern about “compiling drugs into my kid’s system” (Male, age 36). Another described age 11–12 as “way too young,” and age 9 as “extremely early,” and shared they “would rather shots be one at a time than lumped in with other vaccines” (Female, age 35).

Other participants expressed similar concerns about too many shots in one visit. In one group, parents discussed the risk of adverse reactions/side effects if too many vaccines were administered at one time and saw spreading the shots out as a potential diagnostic tool in the event of an adverse reaction: “the number of shots isn’t bothersome. I think it’s just mixing everything that makes or breaks it on my end. That way you can clearly tell if the HPV doesn’t react well or the combo. It’s easier to pinpoint the issues” (Female, age 48). This notion was shared by others regardless of their preferred schedule: “I do like the option of moving the HPV vaccine to the 9 and 10-year-old spot where there is 2 years of nothing” (Female, age 37).

Conversely, two parents valued fewer overall visits that required shots. These participants preferred initiation at ages 11–12 or age 10 equally, noting, “I would rather fewer appointments with shots, even if it means more shots at the appointment” (Female, age 41) and “I don’t like the shots 3 years straight for a kid that old… I take my kids yearly and my son would not like get a shot every year… for 3 years straight” (Female, age 35).

#### 3.2.3. The Parent Follows Provider Recommendations for HPV Vaccine Schedule 

Many participants (n = 9) shared that they largely just deferred to their health care providers’ recommendations about vaccination. Thus, their decisions and preferences rely on the support and/or recommendation of their provider. Participants noted, “If my doctor agreed that the shots should start at 9, that would be fine… The number of shots in a visit or convenience of how often they need to be seen doesn’t matter very much to me, it’s the science behind everything that’s most important” (Female, age 44), and “I accept [initiation at age 9] if it is recommended at the expertise of doctors and scientists” (Female, age 50). However, trust in providers did not preclude participants from expressing hesitancy about age 9 being ‘too young’. For instance, one expressed “9–10 [years of age] feels pretty young. I would be apprehensive… I honestly don’t trust the people in charge of healthcare nationally at the moment, so I’ll stick with our doctor [what the provider recommends]” (Female, age 44). This same participant stated that starting at age 9 was her least preferred schedule, “unless my doctor agreed that shots should start that early. Follow the science and our doctor I trust. Other than that, it doesn’t matter much” (Female, age 44). In this instance, a trusted relationship overrode hesitancy towards initiation at age 9.

## 4. Discussion

The results of this study highlighted mixed preferences among parents for the age at which to initiate the HPV vaccine for their child(ren). In this sample, 47% (n = 20) preferred starting vaccination at age 9, and 49% preferred either initiating at age 11 (n = 12) or age 10 (n = 9). Two participants (5%) had no preference. Three main themes emerged in the qualitative analysis, describing parents’ rationales for adolescent HPV vaccine schedule preferences; these included: the age of vaccination (too young or need for early protection), the number of shots per visit (group together or spaced out), and following health care provider recommendations for vaccination.

Our findings align with another recent research; in particular, quantitative findings from this research team’s national survey (N = 2662), in which parents also had mixed preferences for age of HPV vaccine initiation [21]. In that study, 52.1% of parents rated initiating HPV vaccination at age 11 as the most preferred vaccine schedule, with preference for a younger age of initiation (either 9 or 10) split nearly evenly among the rest of the participants [21].

Our findings related to Theme 1, age 9 is too young, based on concerns about sexual activity and developmental appropriateness, or the benefit of early protection, also emerged as themes in another of this team’s recent qualitative analysis of an open-ended survey item exploring parental perspectives on the age of HPV vaccine initiation [18]. Other research teams have reported similar findings. A recent report of findings from a qualitative study of Spanish- and English-speaking parents noted that English-speaking mothers questioned the need for the HPV vaccine, particularly at the younger age, due to their children’s lack of sexual activity [27]. Both English- and Spanish- speaking mothers expressed concerns about vaccination prior to the onset of puberty possibly interfering with adolescent development [27]. These parental perspectives are in contrast to provider perspectives noted in recent reports, which indicate that providers perceived recommending HPV vaccination at age 9 as beneficial because they believed it would reduce discussion about sexual activity in relation to the vaccine [28,29]. However, in one of these reports, it was also noted that parents themselves were less likely to agree or less ready to discuss HPV vaccine when recommending at age 9 [28]. Moreover, a recently published study of providers found that they reported no change in parental concerns about HPV vaccination or in provider–parent discussion times when recommending vaccination at ages 9–10 compared to ages 11–12 years [15].

In Theme 2, the number of shots per visit/desire to spread out shots influenced parents’ preferences for age of initiation as, in this schedule, starting at age 9 allowed the reduction in the total number of shots per visit. Over the course of the discussions, some parents shifted their preference from age 10 or 11 to age 9 upon realizing that the earlier HPV vaccination allows for fewer shots per visit. Notably, this desire was not limited to those only favoring earlier (age 9) initiation. Spreading out shots was a strong secondary motivating factor noted across stated schedule preferences among the parents. Others have similarly found that health care providers attribute higher parental acceptance of the HPV vaccine before age 11 to two primary factors, one of which is fewer shots per visit [28]. Providers should be aware of these findings as they engage in shared clinical decision-making with parents.

The HPV vaccine has been generally offered at the same time as other routine adolescent vaccines (bundled) in hopes to improve overall adolescent vaccine rates. However, with recent vaccine policy leadership changes, the prior routine adolescent vaccine platform is evolving [30], and providers will need to carefully examine the new recommendations set forth by the CDC as well as other professional organizations (ACS and AAP), and be able to articulate clear rationales for their vaccine recommendations to parents in relation to the evolving routine vaccine recommendation landscape. De-bundling the routine vaccination schedule may have unintended negative consequences which will need to be monitored closely [31]. In a study of Kansas adolescents, the majority who received Tdap in the 6th or 7th grade without concomitant HPV or MenACWY vaccinations did not receive catch-up vaccinations for either HPV or MenACWY within the following two to three years [32]. Further, a study conducted in Oregon highlighted that adolescents that consistently received only one injection per visit were less likely to complete the two-dose HPV vaccine series [33]. Future research should continue to examine parental perspectives (acceptability or hesitancy) as well as biologic and behavioral outcomes of new and varied vaccine schedules, including outcomes related to a potential one-dose HPV vaccine schedule.

Lastly, Theme 3 highlighted that health care provider recommendation remains a sustained and constant driver for parental vaccine decision-making. A strong and positive provider recommendation for HPV vaccination is known to be one of the most important factors associated with vaccine acceptance [34], and is also associated with HPV series’ completion by age 13 [10,11,13]. In this study, those that stated they followed provider recommendation indicated that they would vaccinate at any age per the provider recommendation. In a national survey of primary care providers (N = 1047), 21% reported that they already recommended HPV vaccination at age 9–10, and 61% were willing to do so if routine guidelines changed [13].

Providers must be prepared to educate and support parents in vaccine decision-making, including providing the rationale related to age of initiation for routine recommendations. Strong, clear provider recommendations with a brief evidence-based rationale should be followed by an opportunity for parents to ask questions/engage in further discussion. This is important for trust-building, especially when there are varied guidelines set forth by federal, state, and professional organizations, which may be confusing for parents. Our findings, combined with the results of other research [19], suggest that a strong provider recommendation along with education and discussion may overcome the reluctance of some parents to have their 9–10-year-old children vaccinated against HPV. Additionally, policies that support an expansion of the routine HPV vaccine recommendation (routine from ages 9–12) should allow for flexible vaccine timing. This would accommodate varied parental preferences for age of vaccination and number of shots per visit while still aligning with the public health goal to increase the proportion of youth (over 80%) who obtain the HPV vaccine by ages 13–15 [35].

## 5. Strengths and Limitations/Future Research

Our qualitative study is strengthened by our efforts to recruit nationally; however, the sampling was not nationally representative. While participants were fairly evenly distributed across four of the five US regions, one region (Southwest) did not have any participants. In addition, participants were largely white, educated, and lived in urban or suburban areas. Further, participants were not asked to identify the gender of their child(ren). As with all qualitative research, the findings are not broadly generalizable and should be interpreted as suggestive and hypothesis-generating. There is also potential for selection bias by recruiting participants via a national research panel, as those who sign up for panels may not be representative of the general population of US parents. Future research should target more diverse populations and participants from the Southeast. Despite these weaknesses, the study has notable strengths, as the online text-based focus group methodology has been shown to facilitate active and equitable participation by all participants, particularly when discussing sensitive topics. In addition, the online text-based format allowed parents ample time to review and consider multiple HPV vaccine schedule options, and used visual aids (like a PowerPoint slides) to communicate clearly beyond the text-based discussions. Importantly, this study was conducted in June of 2025, when several major vaccine policy discussions were occurring across the US. Future research is needed to evaluate new vaccine communication strategies, shared decision-making approaches with parents, and outcomes for the evolving vaccine schedules.

## 6. Conclusions

This qualitative study of parental preferences regarding HPV vaccination reveals that US parents have mixed preferences and perspectives regarding the age for HPV vaccine initiation, ranging from 9 to 12 years of age. For those parents who have already made the decision to vaccinate their child(ren) or tend to follow provider recommendations, the age for which the HPV vaccine is recommended by a provider may not impact their overall vaccine behavior. For those parents who have some HPV vaccine hesitancy, recommending at age 9 may increase rather than diminish parental concerns about HPV vaccine discussions involving sexual transmission and sexual activity at that moment in time. Lastly, spreading out vaccine doses may be a motivator for parents to begin HPV vaccination at an earlier age. Given the dynamic vaccine policy landscape in the US, it is essential for providers to understand parental perspectives and motivating factors related to vaccine decision-making, and to integrate these drivers into clinical practice to best support effective communication with families, patients, and communities.

## Figures and Tables

**Table 1:** Three options for when HPV vaccination could be initiated along with other routine adolescent vaccines.

HPV Initiation Age	Age 9	Age 10	Age 11	Age 12	Number of Visits with Shots
(ORANGE) Age 9	HPV	HPV	Tdap; MenACWY		3
(GREEN) Age 10		HPV	HPV; Tdap; MenACWY		2
(BLUE) Age 11 *			HPV; Tdap; MenACWY	HPV	2

HPV = human papillomavirus; MenACWY = meningococcal ACWY; Tdap = tetanus, diptheria, and acellular pertussis. * This was the 2025 CDC/ACIP routinely recommended vaccine schedule at the time of this study.

**Table 2:** Participant characteristics.

Characteristics	Overall
	N = 43
Age	
44 years or less	31 (72.1%)
45 years and older	12 (27.9%)
Race/Ethnicity *	
Non-Hispanic White	27 (64.3%)
Hispanic	7 (16.7%)
Non-Hispanic Black	6 (14.3%)
Non-Hispanic Asian	2 (4.8%)
Sex *	
Female	27 (64.3%)
Male	15 (35.7%)
Education Level *	
High School Graduate	3 (7.1%)
Some College/In College now	9 (21.4%)
College Graduate or Higher	30 (71.43%)
Geographic Area *	
Urban	13 (31%)
Suburban	22 (52.4%)
Rural	7 (16.6%)
US Region	
West	9 (20.9%)
Midwest	11 (25.6%)
Southwest	0 (0.0%)
Northeast	11 (25.6%)
Southeast	12 (27.9%)
Child (age 9–10) already vaccinated for HPV	
Yes	6 (14.3%)
No	37 (85.7%)
Parent of child/ren age 11 or older	
Yes	20 (46.5%)
No	23 (53.5%)
Older child/ren vaccinated for HPV (n = 20)	
Yes	17 (85%)
No	3 (15%)
Parent vaccinated for HPV *	
Yes	14 (33.3%)
No	28 (66.7%)
Parental attitude toward HPV vaccine	
Supportive	32 (74.5%)
Hesitant	7 (16.2%)
Against	4 (9.3%)
How important is it that your child(ren) receive all of their recommended vaccines?	
Not Important	3 (14%)
Important	13 (31%)
Very Important	26 (61.9%)
Preferred age for HPV vaccination (as it relates to the adolescent vaccine schedule)	
Initiate at Age 9	20 (46.5%)
Initiate at Age 10	9 (20.9%)
Initiate at Age 11 (current CDC/ACIP recommendation)	12 (27.9%)
No Preference Indicated	2 (4.7%)

* One participant with missing data.

**Table 3:** Illustrative quotes.

Categories	Illustrative Quotes
Theme 1: A belief that age 9 is too young versus a belief in early protection	
**Too young**	“*I think that [receiving the HPV vaccine at 11–12] is way too young… I think that [a strong immune response] can happen by 15 years… they are still young enough*” (Female, age 35)
	“*I like [the blue] schedule, but it can start at maybe age 13, finish by 16*” (Male, age 36)
	*“I like 10, 11, 12, then their bodies have a chance with each [vaccine], so it can tell if [my child] had negative effects”* (Female, age 48)
	*“Blue [schedule] because they will be more mature [at ages 11–12] and handle the shots better than at 9 and 10”* (Female, age 39)
**Early protection**	*“My understanding is that the vaccine needs to be given prior to being sexually active. I don’t want my kids to be sexually active at, say 13, but I understand that it’s a possibility”* (Male, age 42)
	*“I’m open to the change [to ages 9–10]. As long as it’s safe and the immunity lasts”* (Male, age 40)
	*“If science showed that 10 (or 9, or 11…) were the ideal age, immune system and exposure wide, I would be fine with any schedule”* (Female, age 44)
	*“I only think of [HPV vaccination] in the sense that it won’t potentially hinder their development, why not get the vaccine earlier to get a peace of mind”* (Male, age 40)
**Theme 2: The number of shots administered per visit (a desire to spread shots out or group together)**	
**Desire to spread shots out**	*“I think the body needs to build up antibodies to the vaccine and just like some other vaccines, you can’t push the immune system too much”* (Female, age 46)
	*“I like spreading them out. Better for me and my kids, the less shots per appointment is better for them thus far”* (Female, age 45)
	*“My kids get mad if they get more than 1–2 shots, so occasionally depending on moods, I space them out so I don’t deal with angry tweens”* (Female, age 44)
**Desire to group shots together**	*“I DO think it is always nice to be able to tell the kids ‘there are no expected shots today’ and it is nice to say that for a couple years in a row, it helps them trust their doctor and visits more… maybe for nervous kids”* (Female, age 50)
	*“So, assuming there is no difference in efficacy, I would rather have more shots at one appointment and get them all out of the way”* (Female, age 41)
	*“I’d rather more at once because of how bad my kids hate shots”* (Female, age 34)
	*“My kid doesn’t like shots, so I think more at once is good for us”* (Female, age 37)
**Theme 3: The parent follows provider recommendations for HPV vaccine schedule**	
	*“Orange [schedule] is what my doctor/healthcare provider has recommended, and I trust that they know better than I do”* (Male, age 42)
	*“I accept the change [in vaccination schedules] if it is recommended at the expertise of doctors and scientists”* (Female, age 50)
	*“If it’s 3 [shots] at 11, I’d rather space them out, but ultimately I’d look to my pediatrician, since that’s what we pay him for”* (Male, age 39)
	*“I was just told this at my 18-month-old doctor appointment when I asked [the doctor] if my 9-year-old needed to get the HPV vaccine now, and [my doctor] said my [child] can [get the vaccine] at [their] 11-year-old appointment”* (Female, age 35)

## Data Availability

The data presented in this study are available on request from the corresponding author due to privacy reasons. The raw data supporting the conclusions of this article will be made available by the authors on request.

## Supplementary Materials

The following supporting information can be downloaded at: https://www.mdpi.com/article/10.3390/vaccines14050422/s1, Table S1: Participant characteristics by preferred age for HPV vaccine; Table S2: Primary rationale for parental preferences regarding age of HPV vaccine initiation.

## Abbreviations

HPV: Human Papillomavirus; US: United States; HCP: Health Care Provider.
