# Supplementary material for: Parents’ Preferred Age (9–12) for HPV Vaccination: Decision-Making and Rationale
Source: Vaccines (Basel). 2026 May 7;14(5):422. doi: 10.3390/vaccines14050422 (PMC13211324; doi:10.3390/vaccines14050422)
Supplement: Supplementary file 1 [file vaccines-14-00422-s001.zip › vaccines-4237868-supplementary.pdf]

Table S1. Participant characteristics by preferred age for HPV vaccine.

	Preferred Age for the HPV Vaccine			
**Characteristics**	**Age 9 (n = 20)**	**Age 10 (n = 9)**	**Age 11 (n = 12)**	**Undecided (n = 2)**
Age				
44 years or less	16 (51.6%)	5 (16.1%)	8 (25.8%)	2 (6.5%)
45 years and older	4 (33.3%)	4 (33.3%)	4 (33.3%)	
Race/Ethnicity *				
Non-Hispanic White	15 (55.6%)	4 (14.8%)	7 (25.9%)	1 (3.7%)
Hispanic	2 (28.6%)	2 (28.6%)	2 (28.6%)	1 (14.3%)
Non-Hispanic Black	2 (33.3%)	2 (33.3%)	2 (33.3%)	
Non-Hispanic Asian	1 (50%)		1 (50%)	
Sex*				
Female	11 (40.7%)	4(14.8%)	10 (37.0%)	2 (7.5%)
Male	9 (60%)	5 (33.4%)	2 (13.3%)	
Education Level *				
High School Graduate			3 (100%)	
Some College/In College	5 (55.6%)	1 (11.1%)	1 (11.1%)	2 (22.2%)
College Graduate or Higher	15 (50%)	7 (23.3%)	8 (26.7%)	
Geographic Area *				
Urban	7 (53.8%)	3 (23.1%)	2 (15.4%)	1 (7.7%)
Suburban	12 (54.5%)	4 (18.2%)	6 (27.3%)	
Rural	1 (14.3%)	1 (14.3%)	4 (57.1%)	1 (14.3%)
US Region				
West	5 (55.6%)	2 (22.2%)	2 (22.2%)	0 (0%)
Midwest	4 (36.4%)	4 (36.4%)	2 (18.2%)	
Southwest	0 (0%)	0 (0%)	0 (0%)	0 (0%)
Northeast	6 (60%)	1 (10%)	2 (20%)	
Southeast	4 (33.3%)	2 (16.7%)	6 (50%)	0 (0%)
Child (age 9–10) already vaccinated for HPV				
Yes	5 (83.3%)	1 (16.7%)		
No	15 (40.5%)	8 (21.6%)	12 (32.4%)	2 (5.4%)
Parent of child/ren age 11 or older				
Yes	11 (55%)	2 (10%)	7 (35%)	
No	9 (39.1%)	7 (30.4%)	5 (21.7%)	2 (8.7%)
Older child/ren vaccinated for HPV				
Yes	9 (52.9%)	2 (11.8%)	6 (35.3%)	
No	2 (66.7%)		1 (33.3%)	
Parent vaccinated for HPV *				

Yes	9 (64.3%)	1 (7.1%)	3 (21.4%)	1 (7.1%)
No	11 (39.3%)	7 (25%)	9 (32.1%)	1 (3.6%)
Parental attitude toward HPV vaccine				
Positive	16 (50%)	7 (21.9%)	9 (28.1%)	
Hesitant	3 (42.8%)	2 (28.6%)	2 (28.6%)	
Against				4 (100%)
How important is it that your child(ren) receive all of their recommended vaccines? *				
Not Important			2 (66.7%)	1 (33.3%)
Important	6 (46.2%)	2 (15.4%)	4 (30.8%)	1 (7.7%)
Very Important	13 (50%)	6 (23.1%)	7 (26.9%)	

* One participant with missing data

Table S2. Primary rationale for parental preferences regarding age of HPV vaccine initiation.

Primary Rational				Parental Preference	
**Theme**	**n**	**Sub-Theme**	**n**	**Age for HPV Vaccination**	**n**
A belief that age 9 is too young versus a belief in early protection				Age 9	4
				Age 10	2
		Nine Too Young	15	Age 11	9
				Age 9	3
				Age 10	3
	21	Early Protection	6	Age 11	0
The Number of Shots Administered per Visit				Age 9	8
				Age 10	0
		Spread Out	8	Age 11	0
				Age 9	0
				Age 10	1
	10	Group Together	2	Age 11	1
Parent follows provider recommendations for HPV vaccine schedule				Age 9	5
				Age 10	2
	9	n/a		Age 11	2
